# Preliminary Biomechanical Study of Different Acetabular Reinforcement Devices for Acetabular Reconstruction

**DOI:** 10.1371/journal.pone.0121588

**Published:** 2015-03-23

**Authors:** Ching-Lung Tai, Po-Yi Lee, Pang-Hsing Hsieh

**Affiliations:** 1 Graduate Institute of Medical Mechatronics, Department of Mechanical Engineering, Chang Gung University, Kweishan, Taoyuan, Taiwan; 2 Department of Orthopedic Surgery, Chang Gung Memorial Hospital, Chang Gung University College of Medicine, Kweishan, Taoyuan, Taiwan; Faculdade de Medicina Dentária, Universidade do Porto, PORTUGAL

## Abstract

**Background:**

Acetabular reinforcement devices (ARDs) are frequently used as load-sharing devices to allow allograft incorporation in revision hip arthroplasty with massive acetabular bone loss. The key to a successful reconstruction is robust fixation of the device to the host acetabulum. Interlocking fixation is expected to improve the initial stability of the postoperative construct. However, all commercially available ARDs are designed with non-locking fixation. This study investigates the efficacy of standard ARDs modified with locking screw mechanisms for improving stability in acetabular reconstruction.

**Methods:**

Three types of ARDs were examined to evaluate the postoperative compression and angular stability: i) standard commercial ARDs, ii) standard ARDs modified with monoaxial and iii) standard ARDs modified with polyaxial locking screw mechanisms. All ARDs were implanted into osteomized synthetic pelvis with pelvic discontinuity. Axial compression and torsion tests were then performed using a servohydraulic material testing machine that measured load (angle) versus displacement (torque). Initial stability was compared among the groups.

**Results:**

Equipping ARDs with interlocking mechanisms effectively improved the initial stability at the device/bone interface compared to standard non-locked ARDs. In both compression and torsion experiments, the monoaxial interlocking construct demonstrated the highest construct stiffness (672.6 ± 84.1 N/mm in compression and 13.3 ± 1.0 N·m/degree in torsion), whereas the non-locked construct had the lowest construct stiffness (381.4 ± 117.2 N/mm in compression and 6.9 ± 2.1 N·m/degree in torsion) (*P* < 0.05).

**Conclusions:**

Our study demonstrates the potential benefit of adding a locking mechanism to an ARD. Polyaxial ARDs provide the surgeon with more flexibility in placing the screws at the cost of reduced mechanical performance. This *in vitro* study provides a preliminary evaluation of biomechanical performance for ARDs with or without interlocking mechanisms, actual clinical trial deserves to be further investigated in future studies.

## Introduction

**Fig 1:**
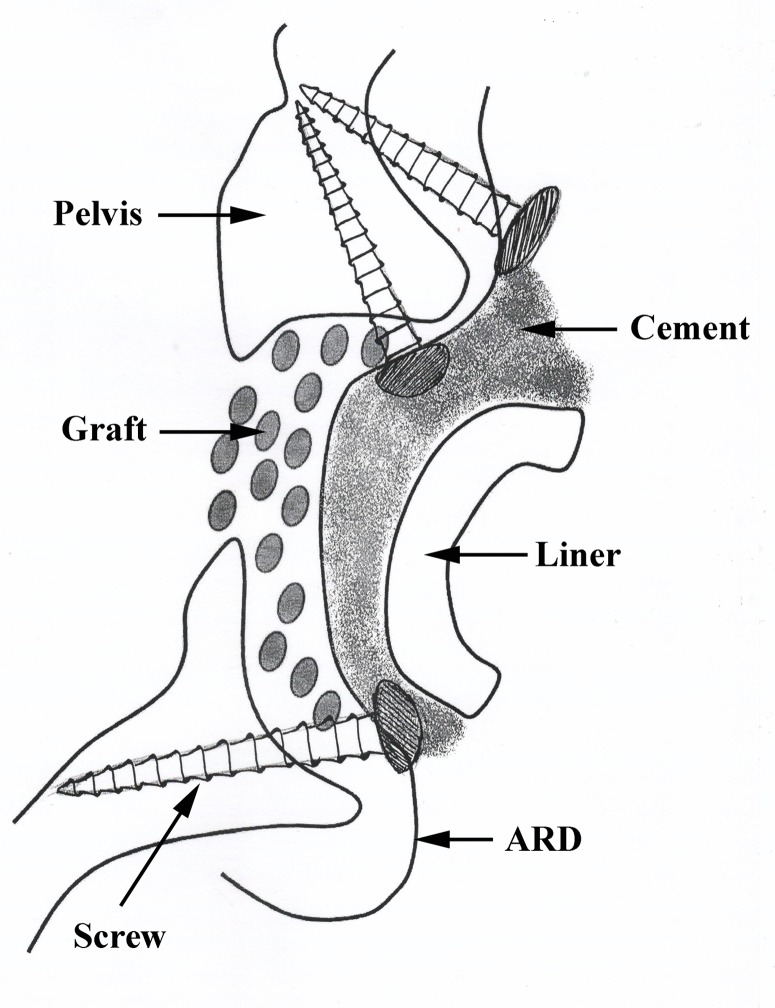
A schematic drawing showing the acetabular reconstruction for a pelvis with a massive acetabular defect. An acetabular reinforcement device (ARD) is used to provide the initial stability of the reconstruction and to allow the incorporation of the allograft into the host pelvis without stress.

Acetabular reconstruction for patients with massive acetabular defects remains one of the most difficult problems in revision total hip arthroplasty (THA) [1,2]. Several factors may cause the loss of acetabular bone, these include the earlier bone removal to accommodate the socket in primary THA, micromotion between the original socket and the pelvis and, lysis caused by wear particles. Acetabular reconstruction for patients with massive acetabular defect makes it difficult to place the new prosthesis in an optimal location biomechanically to achieve sufficient strength for long-term secure fixation. An acetabular reinforcement device (ARD, antiprotrusio cage) is frequently used as a load-sharing device to provide initial stability for the reconstruction and to allow the incorporation of the allograft into the host pelvis without stress [3] (Fig. 1). The application of ARD not only restores the physiological hip function but also prepares the bone stock for future re-revision [1,4,5]. The key to a successful reconstruction is robust fixation of the device to the host acetabulum, which is often very deficient in the revision setting. Inadequate interfacial stability often leads to loosening of the device, absorption of the allograft, and failure of the surgery [6,7].

The stability of the initial fixation of ARDs in revision THA is critical. In particular, it is difficult to achieve stability when the host bone quality is poor or large bony defects are present. Unfortunately, such clinical situations are often encountered in revision procedures [8,9]. The currently commercially available ARDs are all designed with standard (non-locked) screw fixation. The fixation stability of a standard screw mainly relies on the initial screw/bone anchoring strength. As long as the screws are secured, the fixation stability solely depends on the contact friction between the screw head and the implant. The inability of standard screws to resist any external forces acting on the implant in poor-quality bone may create a risk of implant loosening when the contact pressure is low.

**Fig 2:**
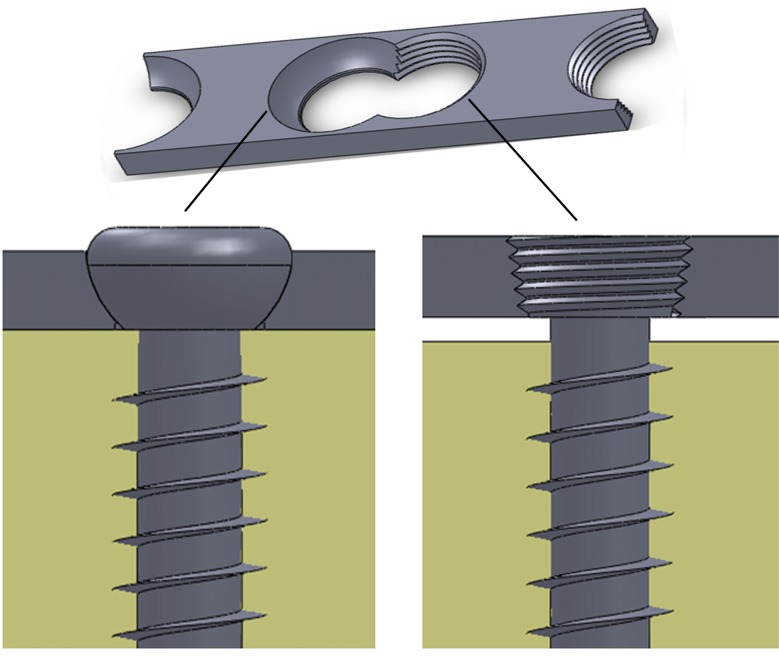
A schematic drawing showing standard compressive screw/plate (left) and monoaxial locking screw/plate (right) systems. For the locking screw/plate system, following the insertion of fixation screws, the screw/plate can be considered an integral device that leads to an improvement in the initial stability at the plate/bone interface.

Locking screw and plate systems with different designs have long been developed for orthopedic trauma surgery. The earlier age of the locking screw/plate devices consisted of monoaxial locking design that have been extensively studied both clinically and biomechanically [10–13]. The monoaxial devices allow for the fixed-angle locking of screws via the threads in the screw head and the plate in an orthogonal manner. The design of these systems results in a rigid screw/plate interface, thereby increasing the force required to displace the construct. Consequently, the monoaxial locking mechanism sacrifices, to some extent, angularity and compression on bone/plate interface to achieve a higher stiffness on screw/plate interface (Fig. 2). The monoaxial locking devices were later developed to allow polyaxial locking, with different fracture patterns and bone structures taken into account [10]. The polyaxial locking system enables the fixation screw to be inserted at a variable angle from the perpendicular aspect. Consequently, the polyaxial locking system allows the surgeon to sense the bone quality and to change the screw direction if required. To date, this new generation of polyaxial locking systems has been widely applied for fracture osteosynthesis [14,15].

Although locking screw systems have been extensively used in trauma surgery to help enhance bone fixation in osteopenic patients, the usefulness of locking screws for fixation of ARDs has not been investigated so far. In the present study, we hypothesized that a locking mechanism for ARDs might provide better initial stability to allow the incorporation of the allograft into the host pelvis without stress. The compression and angular stability of pelvis reconstructed with novel ARDs equipped with either monoaxial or polyaxial locking fixation mechanisms were tested, and their biomechanical properties were compared with those of standard fixation mechanisms. After evaluation of the biomechanical characteristics, this study might provide a preliminary evaluation of biomechanical performance for ARDs with or without interlocking mechanisms. Future investigations such as finite element analysis and actual clinical trial deserve to be conducted in future studies.

## Materials and Methods

### Synthetic bone model

Synthetic composite pelvis (model #3409, Large Right Fourth Generation Composite Hemi Pelvis, Pacific Research Laboratory Inc., Vashon Island, WA, USA) was chosen as testing objects. Synthetic bones are commonly used when imitation of the actual strength properties of real bone is required, and they are suitable for testing, comparing, or designing implants and other devices. Consequently, synthetic bones are suitable for a variety of mechanical experiments when cadaver bones cannot be obtained. To simulate pelvic discontinuity, the standard synthetic pelvis was mounted on a custom-made jig; an oscillating saw was then used to create the standardized transverse gap defect through each acetabulum. By using this method, the level of the transverse osteotomy was kept identical in all specimens.

### Acetabular reinforcement devices (ARDs)

**Fig 3:**
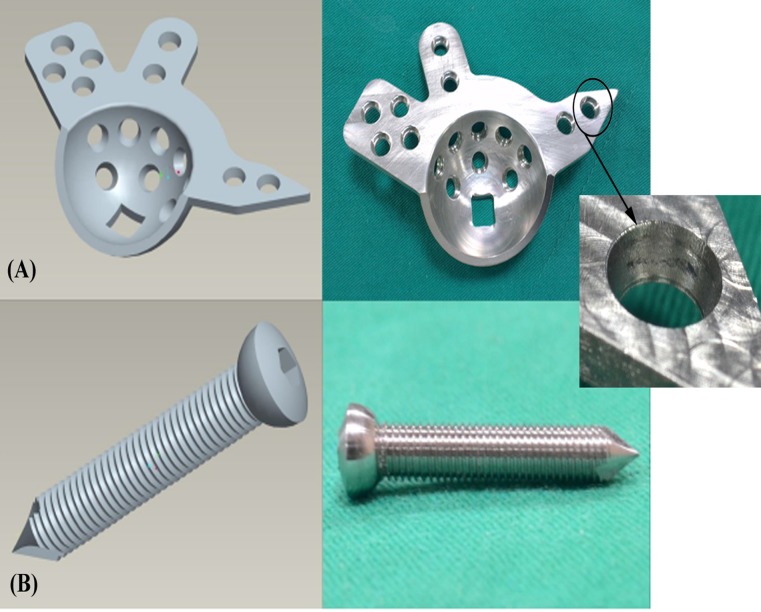
A schematic drawing (left) and a photograph (right) showing (A) a standard ARD and (B) a 5.0-mm compression screw. The screw hole in the ARD is smooth (without threads) and it can be fixed with standard compression screws.

**Fig 4:**
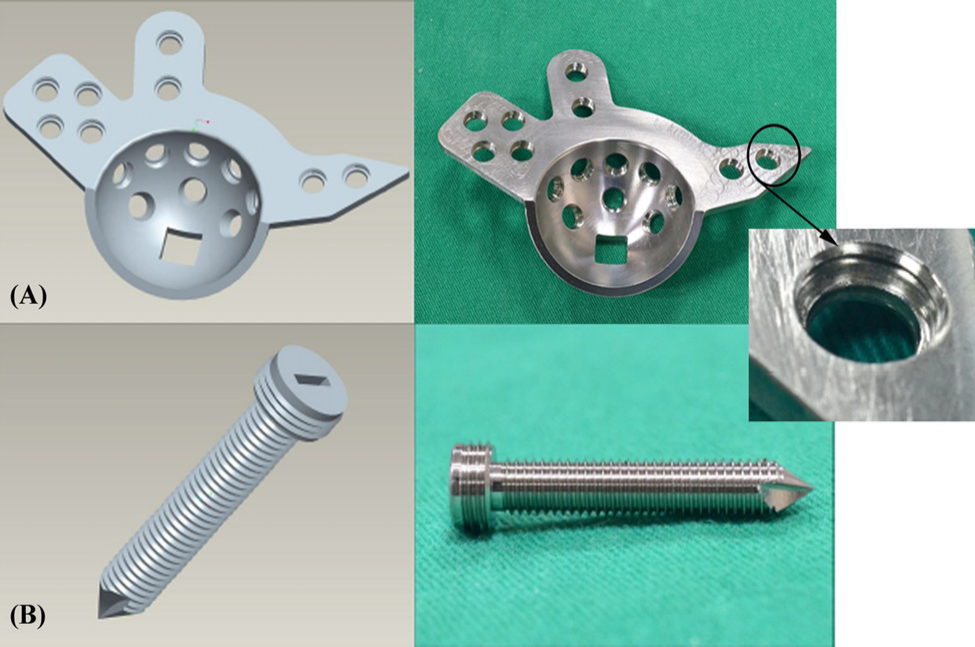
A schematic drawing (left) and a photograph (right) showing (A) a monoaxial locking ARD and (B) a 5.0-mm locking screw. The locking screw has a thread on the screw head, and the ARD has a matching thread. The system allows fixed-angle locking of screws through the fine threads in the head and ARD in a unidirectional manner.

**Fig 5:**
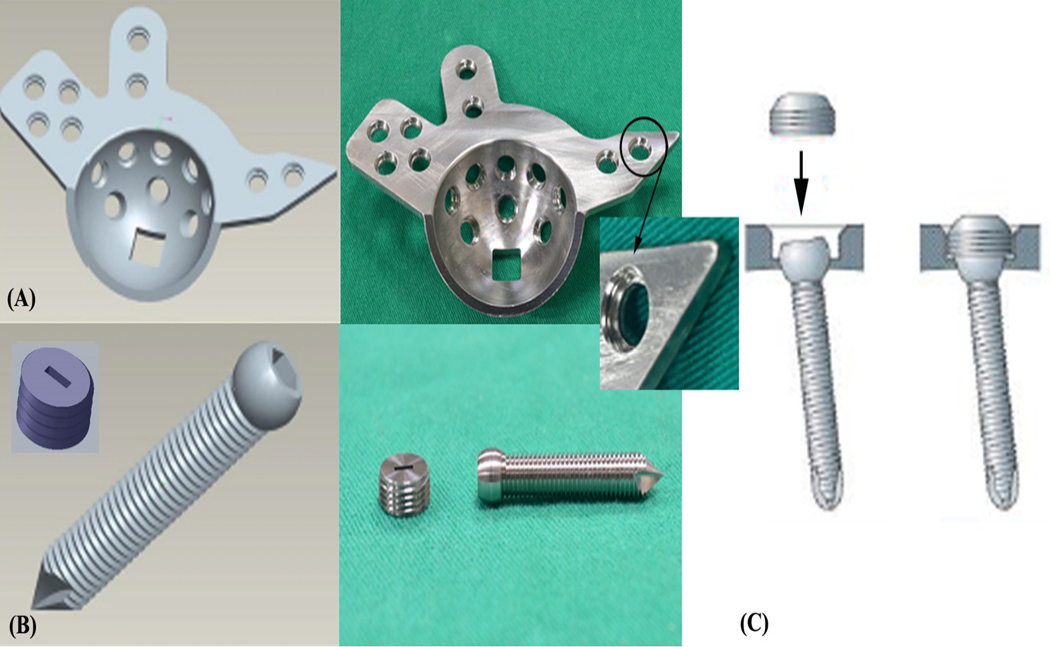
A schematic drawing (left) and a photograph (right) showing (A) a polyaxial locking ARD and (B) a 5.0-mm screw and a locking cap. (C) The system allows the screw to be inserted at a variable angle from the perpendicular aspect and finally locked by a cap. The head of the polyaxial locking screw is contoured to fit congruently into the reciprocal hole, and screw locking is achieved with a locking cap threaded into the screw holes.

Three types of stainless ARDs with a uniform thickness of 3 mm were custom-manufactured using a computer numerically controlled (CNC) machine based on ZCA Acetabular Reconstruction Cages (EDI Code: E1141004, Zimmer Inc., Warsaw, Indiana, USA). The ARDs made of 316L stainless-steel were manufactured with different fixation mechanisms: standard (non-locked), monoaxial, and polyaxial locking. The screw holes of standard ARDs were smooth without threads (Fig. 3A) and fixed with standard compression screws (Fig. 3B). To create the monoaxial locking ARD, the standard ARD was modified with a monoaxial locking mechanism: the head of the locking screw was equipped with a thread, and a matching thread was made in the ARD in a unidirectional manner (Fig. 4). To create the polyaxial locking ARD, the standard ARD was modified with a polyaxial locking mechanism based on the non-contact bridging plate (NCB plate, Zimmer Inc., Warsaw, Indiana, USA). The head of the polyaxial locking screw was contoured to fit congruently into the reciprocal hole, and screw locking was achieved through the use of a locking cap that was threaded into the screw holes (Fig. 5).

The fixation screws used for the three types of ARDs were all 5.0 mm in diameter and 31.0 mm in length, with a thread depth of 0.3 mm and a thread pitch of 0.75 mm. The following three combinations of ARDs and screws were tested: (1) standard ARD attached with standard compression screws tightened to 5.0 N·m (no locking); (2) monoaxial interlocking ARD attached with monoaxial screws tightened to 5.0 N·m (monoaxial locking); and (3) polyaxial interlocking ARD attached with polyaxial screws tightened to 3.0 N·m and locked with a locking cap tightened to 5.0 N·m (polyaxial locking).

**Fig 6:**
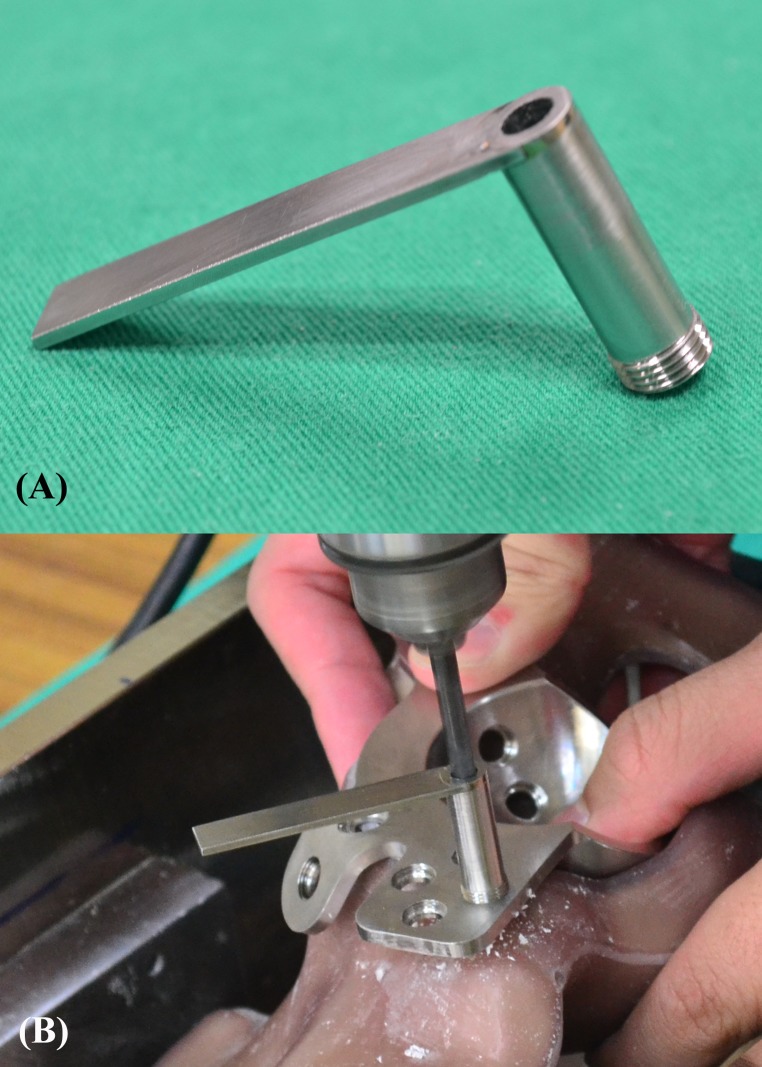
(A) Drilling guide. (B) Drilling guide fixed on ARD for orthogonal drilling of the pilot hole. The drilling guide has a thread matching the ARD thread. It allows fixed-angle placement of screws through the fine threads and ARD in a unidirectional manner.

Each test was conducted on a new synthetic pelvis. The ARD was implanted so that the peripheral flanges were flush with the rim of the acetabulum. All screws were implanted into the corresponding ARDs with orthogonal insertion using a drilling guide (Fig. 6A). The drilling guide had a thread matching the ARD thread. It allows fixed-angle placement of screws through the fine threads and ARD in a unidirectional manner (Fig. 6B). Prior to the insertion of fixation screws, a pilot hole with a diameter of 4.5 mm was drilled orthogonally to the screw hole as determined by the drilling guide. All screws were tightened with a calibrated torque limiting screwdriver set to 5.0 N·m. Following the implantation of the ARD, each specimen was secured in a rectangular metal frame with acrylic (AcryliMet, South Bay Technology Inc., San Clemente, CA, USA) to constrain the movement of ARD during compression or torsion test. The prepared constructs were then mounted on a biaxial servohydraulic material testing machine (Bionix 858, MTS Corp., MN, USA) to compare the relative construct stabilities among the three different types of ARDs. All tests were performed at room temperature (22°C)

### Compression test

**Fig 7:**
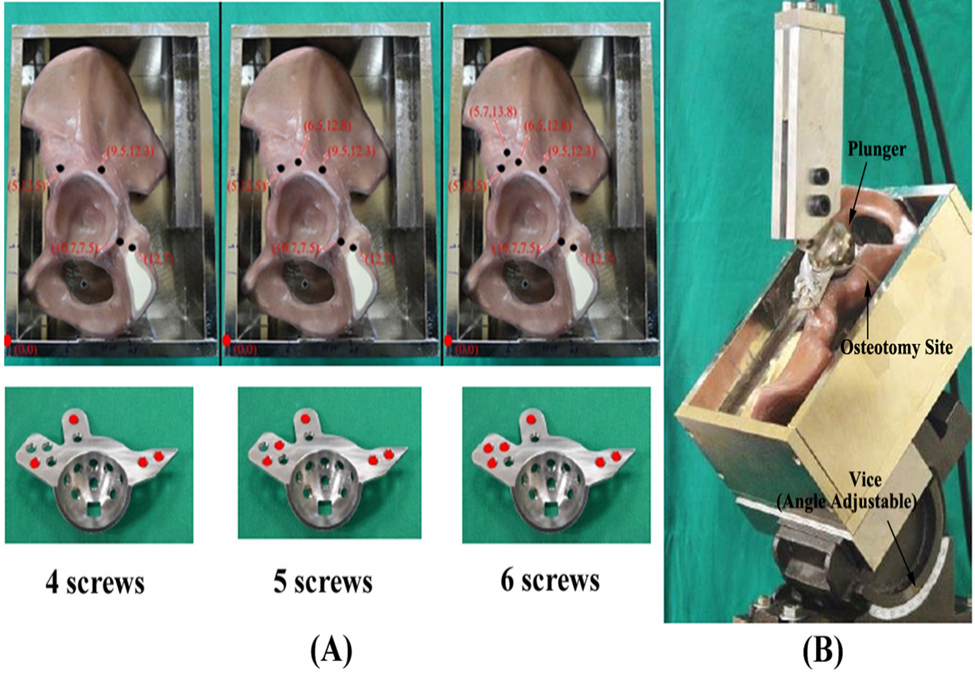
(A) ARDs secured with 4, 5, and 6 fixation screws on the flange of a pelvis. (B) The experimental setup of the compression test. The prepared construct was secured so that the acetabulum was mounted with a 45° inclination on the lower side of the MTS frame and connected to the load cell. An upside-down positioned stem with a 32-mm metal ball was used as the plunger, clamped on the upper side of the MTS wedge grip and connected to the actuator.

A total of 60 pelvises were used (45 for compression and 15 for torsion). In compression, 45 pelvises were divided into three groups (standard, monoaxial and polyaxial). For each ARD group, 15 pelvises were divided into three subgroups with different fixation screw numbers (5 pelvises in each subgroup). Compression stiffness of ARDs secured with different numbers of screws (4, 5, and 6 screws) on the flange of the pelvis was examined (Fig. 7A). Each prepared construct was secured in the rectangular metal frame as previously described. The rectangular frame was then clamped on a custom-made vice capable of angle adjustment, which is connected to the load cell on the lower side of the MTS frame. The angle of the custom-made vice was then adjusted so that the acetabulum was positioned with a 45° inclination (Fig. 7B). An upside-down positioned stem with a 32 mm metal ball was used as the plunger, clamped on the upper side of the MTS wedge grip and connected to the actuator. After positioning the construct, an axial compressive force was applied at a constant crosshead rate of 1 mm/min. The relationship between force and displacement was continuously recorded in 0.1-mm increments (sampling rate: 0.17 Hz) using the MTS Teststar II software. The experimental setup and testing configuration are shown in Fig. 7B.

### Torsion test

**Fig 8:**
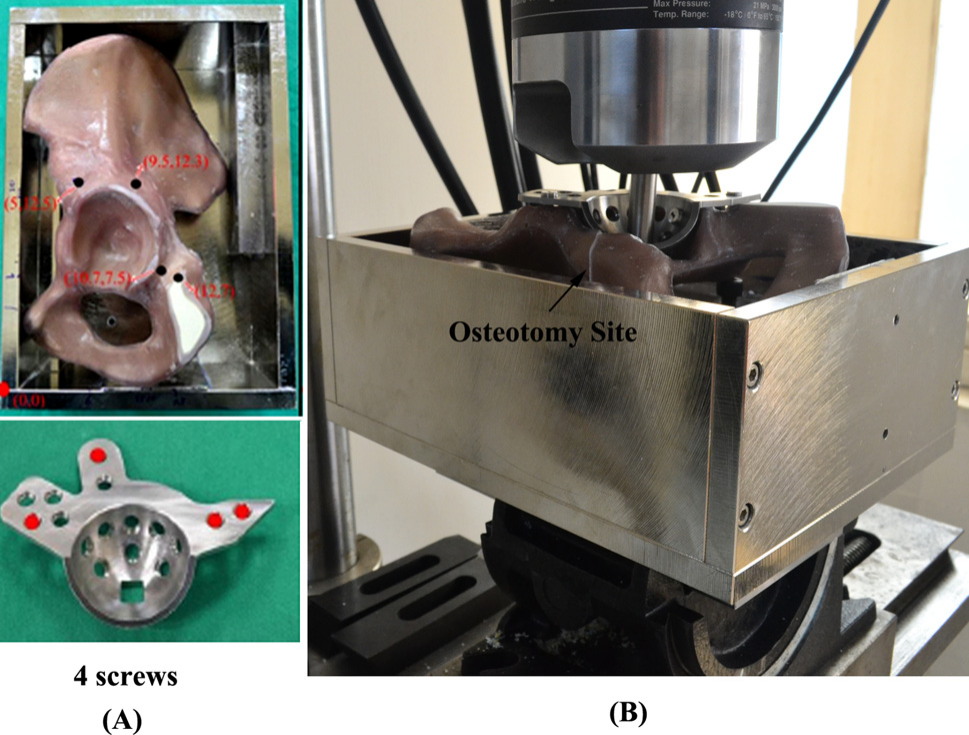
(A) ARDs secured with 4 fixation screws on the flange of a pelvis. (B) The experimental setup of the torsion test. The prepared construct was secured with the acetabulum in a horizontal position. A square bar was used as the transmission shaft; it was clamped on the upper side of the MTS wedge grip and connected to the MTS actuator. For all ARD specimens, a square hole was specially made to prevent the square bar from sliding during the torsion test.

In torsion, 15 pelvises were divided into three groups (standard, monoaxial and polyaxial). All 15 pelvises were secured with 4 fixation screws. Torsion stiffness of ARDs that were secured with 4 fixation screws on the flange of the pelvis was examined (Fig. 8A). Each prepared construct was secured with the acetabulum placed horizontally. A square bar was used as the transmission shaft; it was clamped on the upper side of the MTS wedge grip and connected to the MTS actuator. For all ARD specimens, a square hole was specially made to prevent the square bar from sliding during the torsion test. After the construct was clamped, the torsion test was performed to measure the magnitude of torque by rotating the MTS actuator at a constant rate of 0.1 degree/sec. The relationship between torque and the rotational angle was simultaneously recorded in 0.1-degree increments (sampling rate: 1 Hz). The experimental setup and testing configuration are shown in Fig. 8B.

### Statistical analysis

Means and standard deviations were calculated for descriptive purposes. Multiple comparisons among three different designs of ARD (standard, monoaxial and polyaxial) and different fixation screw number (4, 5 and 6) were performed using a two-way ANOVA test (Minitab 15, Minitab Inc., US), with the significance level set at *P* = 0.05.

## Results

### Compression test

**Fig 9:**
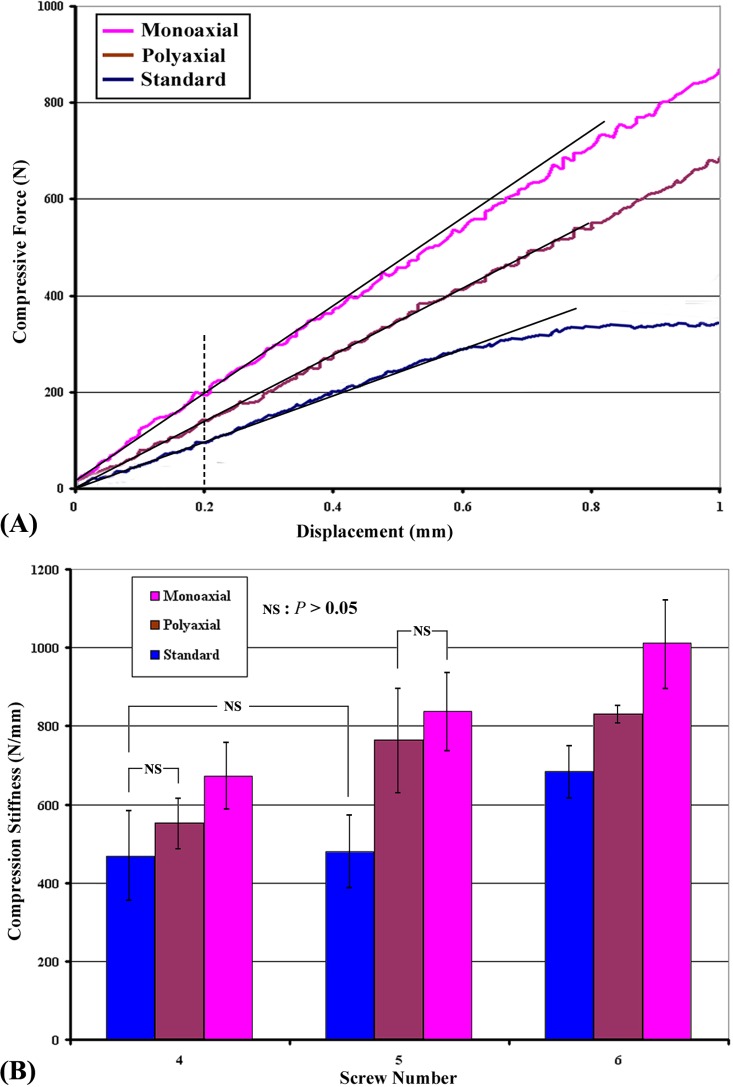
(A) A typical force vs. displacement curve at the initial linear phase of the compression test (0–1 mm). (B) The average compression stiffness of various ARDs secured with 4, 5, and 6 screws determined in the compression test. The monoaxial ARD demonstrated the highest compressive stiffness, whereas the standard ARD had the lowest. The groups without significant differences are indicated with ‘‘NS”.

Fig. 9A shows a typical force vs. displacement curve for the compression test. Compression stiffness was defined as the slope of the curve at the initial linear phase (straight line passing through the value of force at 0.2 mm in displacement). The mean compression stiffness of various ARDs secured with 4, 5, and 6 screws is shown in Table 1 and Fig. 9B. The results indicated that, regardless of the ARD type (monoaxial, polyaxial, or standard), the compression stiffness increased with increasing screw number, the exception was ARDs secured with 4 and 5 screws in the standard group (*P* > 0.05). Additionally, the monoaxial ARDs with the same fixation screw numbers (4, 5, or 6) had the highest stiffness, whereas the standard ARDs had the lowest. The exceptions were that no significant difference was found between 4-screw ARDs of the standard and polyaxial groups (*P* > 0.05), and 5-screw ARDs of the polyaxial and monoaxial groups (*P* > 0.05).

**Table 1:** The average compression stiffness and torsion stiffness of various ARDs secured with different screw number. Torsion stiffness of ARDs without screw number of 5 and 6 are indicated with “NA”.

ARD Types	Screw Number	Compression Stiffness (N/mm)	Torsion Stiffness (N·m/degree)
Standard	4	381.4 ± 117.2	6.9 ± 2.1
	5	487.4 ± 92.4	NA
	6	684.2 ± 65.5	NA
Polyaxial	4	552.8 ± 62.8	11.4 ± 1.3
	5	765.5 ± 132.4	NA
	6	832.7 ± 24.2	NA
Monoaxial	4	672.6 ± 84.1	13.3 ± 1.0
	5	838.6 ± 99.8	NA
	6	1,012.1 ± 111.2	NA

### Torsion test

**Fig 10:**
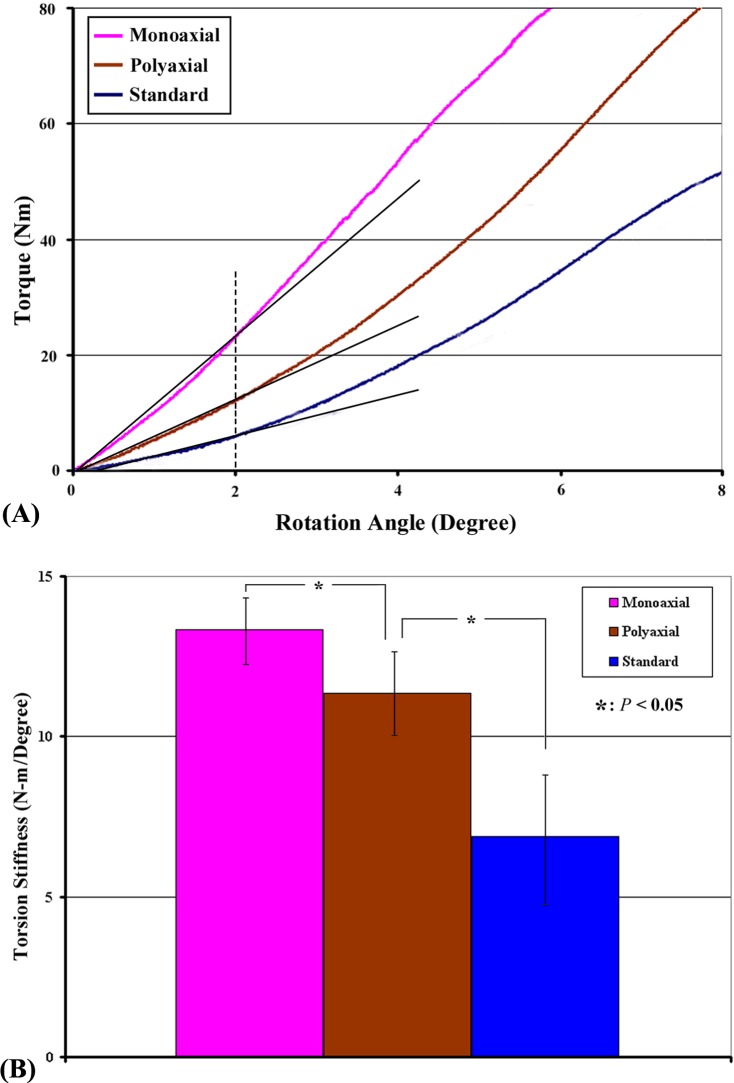
(A) A typical torque vs. angle curve at the initial linear phase of the torsion test. (B) The average torsion stiffness of various ARDs secured with 4 screws determined in the torsion test. The monoaxial ARD demonstrated the highest torsion stiffness, whereas the standard ARD had the lowest. Significant differences in torsion stiffness were found among the groups (*P* < 0.05).

Fig. 10A depicts a typical torque vs. rotation angle curve for the torsion test. Torsion stiffness was defined as the slope of the curve at the initial linear phase (straight line passing through the value of torque at 0.2 mm in displacement). The mean torsion stiffness of various ARDs is shown in Table 1 and Fig. 10B. Significant differences in torsion stiffness were found among three groups (*P* < 0.05). The stiffness of the monoaxial locking construct (mean value: 13.3 ± 1.0 N·m/degree) was 1.18 times higher than that of the polyaxial locking construct (mean value: 11.4 ± 1.3 N·m/degree) (*P* < 0.05). The stiffness of the polyaxial locking constructs was 1.65 times higher than that of the standard non-locked constructs (mean value: 6.9 ± 2.1 N·m/degree) (*P* < 0.01).

## Discussion

Revision THA with severe acetabular bone loss is a reconstructive challenge. Clinically, the most common treatment for acetabular bone loss in revision hip arthroplasty is bone grafting with a bulk allograft that is then stabilized with an ARD. Previous studies recommend using ARDs to protect bulk bone grafts in load-bearing defects [16–18]. The use of an ARD is proposed to improve osseointegration because it protects the underlying graft from excessive mechanical stress while supporting the cup, restoring the limb length, and maintaining better bone stock for future revision [4,5]. Unfortunately, the use of allografts has shown poor clinical outcome [19–21]. The failure of the allograft treatment may be attributed to inadequate fixation and excessive stress on the allograft [22,23]. Initial stability of the ARD is crucial for improving the survival rate because excessive motion at the bone/implant interface may result in the eventual failure of osseointegration, which reduces the success rate of acetabular reconstruction [22,23].

Extensive studies have described the mechanical roles of ARDs in revision THA [24,25]. However, studies addressing the effectiveness of load sharing mechanisms using the concept of locking mechanisms are lacking. According to our results, the standard compression screws exhibited the lowest construct stability in terms of resistance to compression and angular forces. Standard compression screws achieve their fixation based solely on the initial screw/bone anchoring strength. When these screws are secured, they rely on contact pressure between the screw head and the implant to resist external translation and angulation. The compression load from the screw head is transferred only to the bone that is engaged by the screw threads [26]. Consequently, standard compression screws are incapable of producing significant resistance to any external forces acting on the implant in poor-quality bone. The application of standard compression screws in ARD can cause a severe problem in cases with poor bony purchase secondary to either poor-quality bone or a large defect. In contrast, the results of both the compression and torsion tests revealed that the monoaxial locking mechanism exhibited the highest acetabular construct stability; therefore, it provides the most robust support for the acetabular socket and allows underlying bone grafting in an environment that is protected from excessive stress. However, the monoaxial locking screws were inserted in a unidirectional configuration, which may restrict the ability of the surgeon to determine screw placement. This can result in a problem when there is either poor-quality bone or a large defect present in the screw placement path. Our results indicated that the polyaxial locking screws exhibited construct stability intermediate between the monoaxial and standard screws. The polyaxial locking screws were considered beneficial because they allowed screw insertion at a variable angle while providing compression in the same manner as a standard screw prior to insertion of the locking cap. The use of polyaxial locking screws might resolve the problem of determining the screw insertion angle in relation to good-quality host bone.

Locking screws have widely been used in orthopedic trauma surgery but have had limited application in arthroplasty surgery. In the authors’ opinion, in contrast to ARDs in acetabular reconstruction, the interlocking mechanism is inappropriate for fixation of the acetabular cup in primary cementless THA. For cementless THA, compression is necessary to maximize the contact between the bone and the cup, which increases the friction between the two surfaces and thereby potentially facilitates bone ingrowth. In such cases, the application of an interlocking mechanism between the screw and the cup would increase the screw/cup interfacial stability. However, this mechanism causes an enormous reduction of bone/cup interfacial pressure. In extreme conditions, the screw locking mechanism may even create a gap between the cup and the bone, leading to the failure of bone ingrowth into the acetabular cup.

Numerous reports utilizing synthetic pelvis to investigate the mechanical stability of postoperative pelvis for acetabular reconstruction have demonstrated that the synthetic pelvis is a good alternative for in vitro experiments when human pelvis cannot be obtained [25,27]. Gililland et al. [25] used synthetic pelvis to evaluate the mechanical stability of three types of acetabular reconstruction constructs: a cup-cage construct, a posterior column plate construct, and a bicolumnar construct. Their results demonstrated that the bicolumnar construct provided improved component stability. Recently, Milne et al. [27] compared polyaxial compression locking screws with non-locked and cancellous screw constructs for acetabular cup fixation using synthetic pelvis. Their results revealed that polyaxial locking compression screws significantly improved the construct stiffness compared to non-locked or cancellous screws. They concluded that the increase in construct stiffness will likely reduce interfacial micromotion. In the present study, the synthetic pelvis was chosen as a substitute for human pelvis because of the difficulty of obtaining human pelvis and reliability of results acquired using specimens with uniform properties.

Our study has limitations. First, the ARDs were manufactured in house using only one type of metal, shape, and surface finish. Possible effects of variations in the above-mentioned properties were not considered. Second, a synthetic pelvis was used as a substitute for human bone. Although synthetic pelvis provides a platform for comparison of fixation stability, there must be some differences in mechanical characteristics between the synthetic pelvis and actual bone; therefore, extrapolation of our results for clinical application should be done with caution. Third, the evaluation of compressive and angular stiffness did not account for the surrounding muscle forces, which may impact the clinical relevance of the results. However, we believe that the results of compressive and angular stiffness provide a preliminary platform for comparison of the postoperative stability of ARDs with various locking designs in acetabular reconstruction. Fourth, a square bar was used as the transmission shaft for torsion test. During torsion test, the angular distortion of the square bar might affect the measured torsion stiffness of the ARD construct. However, torsion stiffness of all ARD constructs were tested in a reproducible manner using an identical stiffed square bar, and we believe that this study provides a comparison of the mechanical performance of various ARDs in an artificial pelvis. Fifth, this was an in vitro analysis of specimens prepared in a laboratory environment, which did not take into account the effects of temperature and body fluids on compression and torsion tests. Finally, only static loading (compression and torsion in the synthetic pelvis) was conducted without consideration of other types of physiological loading. In real-life situations, the screw/bone, screw/implant and implant/bone interfaces are subjected to dynamic multi-directional loading. Although our loading mode does not necessarily represent the actual physiological loading conditions, all specimens were prepared and tested in a uniform and reproducible manner, and we believe that this study provides information that can be useful to orthopedic surgeons performing acetabular reconstruction. Further investigation of the effects of other loading methods, such as dynamic fatigue testing, might be necessary in the future.

## Conclusions

Equipping ARDs with interlocking mechanisms effectively improves the initial stability at the device/bone interface compared to standard non-locked ARDs. Our study demonstrates the potential benefit of adding locking mechanisms to ARDs. Monoaxial ARDs provide the most robust support for the acetabular socket. However, the monoaxial locking screws are inserted in a unidirectional configuration, which may restrict the ability of the surgeon to determine screw placement. Polyaxial ARDs provide the surgeon with more flexibility in placing the screws at the cost of reduced mechanical performance. This *in vitro* study provides a preliminary evaluation of biomechanical performance for ARDs with or without interlocking mechanisms, future investigations such as finite element analysis and actual clinical trial deserves to be conducted in future studies.
